# Deliberation, Citizen Science and Covid‐19

**DOI:** 10.1111/1467-923X.12869

**Published:** 2020-07-11

**Authors:** Harry Pearse

**Keywords:** deliberative democracy, citizen jury/assembly, citizen science, Covid‐19

## Abstract

Rather than aiming to produce more ‘rational’ or more ‘other‐regarding’ citizen judgements (the outcome of which is uncertain), deliberative democratic exercises should be re‐designed to maximise democratic participation. To do this, they must involve citizens *and* experts, a novel arrangement that will benefit both cohorts. For the former, a more inclusive form of deliberation will offer an opportunity to contribute to political discussion and be listened to by people with political or policy‐based authority. For the latter, it will provide a venue through which expertise can be brought to bear on democratic decision making without risk of scapegoating or politicisation. More broadly, deliberation that prioritises dialogue (over, say, opinion change) affirms the principle that political decisions reflect value judgements rather than technically ‘right’ or technically ‘wrong’ answers—judgements that are legitimate if arrived at through discussion involving the people due to be affected by the resultant policy. This article sets out the advantages of this form of deliberation—which bears some similarity to certain types of citizen science—in the context of the UK government’s responses to Covid‐19; both the confused decision making evident to date, and the forthcoming re‐opening phases that will prioritise or advantage some constituencies over others.

## Introduction

during a public health crisis in which scientific and medical expertise is paramount to decision making, what is the role or function of democratic actors and institutions? The question itself signals a recent shift in political sentiment. Disparaging remarks about experts ‘from organisations with acronyms’ seem like figments of a bygone age. The UK government now says it’s ‘following’ the science (compiled and relayed by its advisory group, SAGE, and its subcommittees, SPI‐M and SPI‐B, as well as outside bodies like the London School of Hygiene and Tropical Medicine) and, to date, its Covid‐19 measures have received very little legislative or public scrutiny—a bill rushed through Parliament; minimal or no citizen engagement. The answer, therefore, seems to be: very little, or no role at all.

At the same time, it remains unclear exactly how democratic governments should deal with expert knowledge. Following is preferable to ignoring or disregarding. But total deference discounts the fact that science or expertise is usually uncertain and rarely consensual. It also allows politicians to escape responsibility and blame experts when things go wrong. An alternative would be to formally integrate experts into representative politics itself—to make them political actors alongside all the others. This, however, would compromise experts’ political impartiality, as the Prime Minister and his Chief Scientific Advisor and Chief Medical Officer argued when the latter were quizzed about the behaviour of Dominic Cummings.

The actions taken by the government in response to the disease—and their baffling attempts to balance these considerations—are familiar. Though an outlier to begin with—delaying the peak rather than flattening the curve—the government soon had second thoughts (closing schools, then pubs and restaurants), before lurching from mitigation to suppression proper on 23 March 2020. Only when lockdown was introduced did the measures apply to more or less everybody. At which point—although some communities and businesses have and will continue to suffer more than others—in principle the measures affected us in the same way and to the same degree.

This ostensible equality, the emergence of what seemed like (or was presented as) a unified scientific view, plus the obvious urgency of the measures themselves, partly explain why the public and the political establishment have so far been content to abide by, and only minimally contribute to, these—by all standards—monumental decisions.

However, nothing lasts forever, and there are few things as conventionally short‐lived as political sanguinity (cue the polls showing waning public satisfaction with the government’s handling of the pandemic). So, as the next phase of the Covid agenda begins, and as society and the economy start to open up—to varying degrees, at different rates, for some people at one time, for other people at another—the need for public consent (and democratic legitimacy) will surely increase.

This is a straightforward democratic calculus. When politics mandates clear winners and losers—directly, rather than by implication—public buy‐in is critical. Citizens must partake in or give consent to decisions that restructure their communities and possibly divide them. The Cummings affair gave notice that such imbalance is possible. And decisions that formally entrench social division are heading our way. Immunity passporting, for example, will drive a wedge between those allowed to travel or work and those who aren’t, and it’s hard to imagine a scheme that creates temporary forms of tiered citizenship being implemented without ironclad democratic legitimation measured by public acceptance.

Lurking beneath these conundrums are deeper questions about the functionality of representative democratic infrastructure. What purposes do these institutions serve? Are they meeting their own democratic standards, even in normal times? And how might the system be improved or adapted? This article offers some preliminary answers, using the government’s Covid measures as a backdrop for understanding the political assumptions and choices that underpin democratic institutional design. I start with a critique of representative democracy, and an outline of the ways in which deliberative democracy might address its shortcomings. I go on to argue that these ideas about deliberation are flawed, and that deliberative exercises should be reconceived to prioritise their inclusive, rather than epistemic, qualities. The basis of this re‐design is to include lay people *and* experts in deliberation. I conclude by comparing this form of deliberation to certain types of citizen science, arguing that, as well as their potentially useful role in shaping the still‐evolving Covid agenda, these mechanisms ought to be permanent features of the UK’s democratic landscape.

## Deliberative democracy

There is plenty of evidence that representation isn’t living up to the billing: low voter turnout, a transparently partisan media, the recrudescence of populism, and the corruption or hollowing out of liberal institutions. These are symptoms of something gone awry. But the problem, at root, is that representative democracies are not fulfilling their basic conceptual promises. There are various ways this might be true. And how one frames the problem inevitably conditions the nature of the possible solutions.

A credible and oft‐touted antidote to our democratic failings is the use (and regularisation) of more deliberative forms of democracy—usually citizen juries or assemblies.^1^ Experiments in deliberation are taking place across the world—in Dublin, in Oregon, in Porto Alegre—and their generic features are now familiar. Small groups of representatively sampled citizens, briefed by experts, given the time and space and a professionally moderated discursive environment to talk about political questions of significance or controversy. These are broad parameters. But deliberative exercises vary in terms of organisational structure, as well as the goals we set for them. It all depends on which problems in the representative system we think deliberation should address.

For example, we might work from the premise that representative democracy fails to ascertain and implement the common good, perhaps owing to the short‐term, sectional incentives introduced by elections. Or, accepting a more fractious, competitive view of democracy, we might complain (still) that citizens are too uninformed or uninterested in politics to formulate coherent sectional interests. Many deliberative theorists and practitioners make precisely these assumptions.^2^ And, therefore, they theorise and design deliberative events to counteract these deficiencies.

Usually, then, the immediate objective of an ‘ideal’ deliberation is to produce informed opinion (in light of expert briefings), strengthened by rational challenge, and moderated by an appreciation of the interests and concerns of the other participants. If the deliberations yield consensus, the recommendations can be construed as a counterpoint to short‐term electoral politics, and a claim about the common good is plausible.^3^ If not, the rules and boundaries of the deliberative process will ensure individuals’ own opinions are more coherent, and that the marketplace of ideas is able to function properly.^4^ Provided the jury or assembly is formally connected to the representative system—by, say, a government commitment to put its recommendations to a referendum—deliberation can be said to fulfil the original legitimacy requirement; that citizens in democracies participate in the decisions that affect them. This may seem like a big ‘if’, but a similar pledge was made by the French government vis‐à‐vis its recently completed Climate Convention.

On paper, then, this model looks great. It pulls off the double feat of strengthening the democratic bond between government and citizens *through* a process of improving the decision‐making capacities of the latter. Win‐win. In practice, however, things are more complicated. The issue is the second part of the proposition—the idea that deliberation produces more rational or other—regarding decision making. This is by no means a given, a problem drawn out in the work of Yale professor of psychology, Dan Kahan.^5^ Using the backdrop of climate change—often cited as a subject in need of deliberative scrutiny—and drawing on extensive American survey data, Kahan and his colleagues reveal that public concerns about climate do not correlate with scientific knowledge or reasoning capacity. That’s to say, it’s not true that American citizens who know more about science and score higher on reasoning tests worry more about the risk of climate change than their less well informed, less rational peers. In fact, the data show that the more educated and rational people are, the more they are polarised on particular (usually large and diffuse) political questions, like climate.

These are startling findings, and, for certain issues, they pose a serious challenge to a deliberative model that relies on people’s minds being changed by exposure to new and better information or participation in rational discussion. This and other work in social psychology and science communication does not invalidate that aspect of the deliberative proposition to do with creating more direct connections between citizen concerns and government policy. But it should prompt us to reconsider how the events themselves are structured. They must—in some cases—be geared less towards opinion change, and focus more on what they can realistically achieve: political equality and inclusion.

This places deliberation in a new and different relationship to representation, prioritising engagement with its social rather than epistemic limitations. In this guise, deliberation addresses the critique that, despite our nominal political equality (one person, one vote), political participation is overwhelmingly the preserve of particular constituencies (the old, the rich, and the white), and that social divides (in age, in education, and in location) are distorting the democratic map. So, instead of holding deliberative exercises to generate and ascertain a ‘best case’ or ‘most rational’ public perspective on a particular issue, we design them to maximise the *experience* of democratic participation: opening up—to as broad a range of people as possible—the opportunity to participate, contribute and be heard by others.

I think that the best way to do this is to include lay people *and* experts in deliberation. To get ordinary citizens and subject specialists or bureaucrats to engage in open and equal conversation with one another, both parties receptive to the value and insight brought to the table by the other. It’s a simple idea (if not straightforward to execute), with potentially far‐reaching consequences. However, in a political context, it’s never, or only very rarely, been tested.

In conventional deliberative events, like citizen juries, experts are called upon to brief the lay participants and field questions, but they do not participate in the deliberations themselves. There are several reasons why this might be the case, but it broadly makes sense given what most deliberative events are set up to achieve. If the point of deliberation is to yield an optimal, or most rational, public judgement on a particular issue, it stands to reason that the role of experts is simply to inform and enlighten—to create conditions in which lay participants can improve their judgements. This dynamic changes, however, if the priority of deliberation is inclusion. Then, it makes sense for experts themselves to participate, for knowledge to be passed in both directions—from experts to citizens, but also from citizens to experts—and for *both* parties to assume they can learn something from the other.

In a sense, this will narrow the (social and epistemic) gap between experts and citizens, which may seem like a reduction in the authority of the former. In reality, however, both parties can potentially benefit. For citizens—particularly those unaccustomed to democratic participation—more inclusive, more dialogic forms of deliberation might be a rare, perhaps the first, chance to participate in political discussion and decision making. An opportunity to be consulted, not just by their peers, but by decision and policy makers; people with authority. It’s hard to know for certain how this will affect their sense of political agency; whether or not it will incline them to further democratic participation. But, conducted properly and in good faith, these exercises can demonstrate to people otherwise overlooked by democratic politics that they have political value and meaning.

As for the experts, ensuring they are represented in deliberation will provide insurance that policy will be informed by expertise, without creating a situation in which experts are entirely culpable for mistakes, or overly politicised by their involvement in decision making. Party politics tends to intrude between expertise and policy design, or it did in the pre‐pandemic world. Involving experts in deliberation will ensure, post‐pandemic, as party politics reasserts itself, that expertise receives a fair public hearing and is in some respect brought to bear on questions of policy. (The value of mechanisms that openly give voice to expert opinion is becoming clear again as signs emerge that the UK government is pushing back on certain expert recommendations ‐ distancing rules, for example).

On the flip side—and particularly important now, when political judgement is still largely deferring to, or hiding behind, expert instruction—structuring deliberation so it involves a *range* of expert voices, as well as a variety of potentially countervailing citizen concerns, will help counteract the technocratic assumption that objectively ‘right’ answers exist and that it is simply the job of politics to uncover them. This assumption leads to power being concentrated in the hands of those who apparently know ‘best’, the cohort technically trained to uncover the answers; experts not citizens. Challenging this assumption, and accepting that political questions and answers always reflect value‐judgements and conflict, and that they’re resolvable (at least potentially) through discussion and debate, reasserts the principle that people ought to have a say over the decisions that affect them.

The upshot is policy that, while guided by expert judgement, reflects—or at least attempts to reflect—the interests of a cross‐section of citizens. It may or may not produce the most ‘rational’ or the most technically efficient outcome. But if experts are open to and cognisant of lay concerns and priorities, they will design policy that is more consensual, more practicable, and therefore seen to be more legitimate.

This is precisely the course that policy makers will need to take as they embark on the next round of Covid measures. It won’t be enough to simply follow the science—a problematic proposition in itself. The advice of epidemiologists, immunologists, social scientists, and psychologists, must of course be sought and in many cases heeded. But the collective response, whatever it is, will reflect a series of value judgements about, among other things, liberty and security, right and obligation, risk analysis and the value of life—and how they are measured. ‘Science’ can’t resolve these judgements, at least not fully. But they pose questions that every member of a political community has a right, and the capacity, to help answer.

## Regular and citizen science

This reference to ‘science’ is actually shorthand for a particular conception of science. It’s a prominent conception, but not the only one. In fact, in some respects, the differences between the government’s attitude to science and expertise, and the reconfiguration of the politics‐science, citizen‐expert relationship that I’m proposing, reflect a growing distinction within scientific practice itself and the discipline of science communication. This is the distinction between ‘regular’ science and citizen science.

Since the Second World War, the scientific enterprise has largely (and not always unreasonably) sought to identify with the regular conception of science. That is, as a self‐governing and value‐neutral activity, independent of politics.^6^ In this model, experts are often highly specialised and apolitical. And in its most technocratically‐minded formulation, the scientific expertise is a way of guiding and setting the boundaries for the value problems of politics.^7^

Of course, the model has never been perfectly realised, and peer‐review scandals, technical failings, as well as the science community’s closeness to either business or environmental groups, have undermined claims that science straightforwardly speaks truth to power.^8^ Nevertheless, if we take seriously the oft‐repeated ministerial assertion that government is simply, and perhaps helplessly, heeding expert guidance, we *are* in some sense living in a world structured according to the regular science view of the scientific establishment. An entity outside the wrangling of politics, mandated to provide instruction for politicians and citizens.

There are several outwardly and genuinely credible aspects to this perspective. Science is supposed to be a dispassionate activity, and scientists, in their capacity as scientists, are not partisan in the same way politicians are. To some extent, then, science can and does stand apart from politics. Its drawback, however, is an oversimplified account of the nature and production of knowledge.

Contemporary understandings of knowledge, including scientific knowledge, recognise that information is, or can be, socially or culturally influenced.^9^ It’s common, for example, for knowledge claims or modelling projections to be based on highly contextual and contested notions of risk and value.^10^ Political contingency—and certainly the appearance of it—is therefore a possibility. Even when knowledge isn’t put to use, it’s almost always sought for a specific purpose. The objects of scientific inquiry, and the use they’re put to (if indeed they are), are therefore embodiments of particular values. And science *advice*—the result of knowledge from different disciplines being bundled together—is very often uncertain and decided upon by politicised regulatory committees.

Covid science is a case in point. The models produced by both the London School of Hygiene and Tropical Medicine and Imperial College London are not products of disinterested data collection and analysis, but exercises tailored to address concrete, real‐world problems. The latter started out as a model to assess responses to one thing (avian flu in Thailand) and was re‐purposed to assess another (coronavirus in the UK). Nor do the models convey discrete and uncomplicated epidemiological truths (or other disciplinary insights). Some of the data are epidemiological, but some were sourced or produced using other tools or disciplines, including estimates about the number of people an infected person is likely to come into contact with. This is just one of a number of estimates, derived from imperfect samples, building uncertainty into the modelled outcome.

Whenever knowledge is uncertain or contingent, it raises important questions about who is accountable for its acquisition and implementation, and on what basis. To a degree, it undercuts the notion that knowledge—credible knowledge—is or must be sought from experts or expertise. Some conceptualisations of *citizen* science build on these assumptions; broadening notions of expertise and engaging the issue of accountability by introducing ordinary people into the processes of making, directing or assessing knowledge‐claims.^11^ The types of activity encompassed in these conceptions are vast, and can be as light touch as, say, contributing observations to ornithological studies, or as involved as helping to devise and target a research programme into air pollution. They don’t exclude ‘conventional’ experts; they are forms of expert‐lay collaboration. But underlying this particular subset of citizen science activities is the idea that people are both capable of contributing to scientific knowledge, and that they have a right to participate in knowledge production that will in some way affect them.

This is what makes them similar—in terms of structure and objective—to inclusive, dialogic forms of deliberation. Collaborative citizen science has received more theoretical attention, and practical testing, than this type of deliberative democracy. The latter, however, has a potentially wider—that is, non‐scientific—application. But the former offers a useful template for how experts or scientists can be integrated into decision‐making processes (with citizens) without surrendering their intellectual integrity or suffering reputational damage.

## Covid science

These principles have a bearing on the government’s ongoing Covid agenda. To be clear: the response must continue to be guided by expert research. But it should also be responsive to the array of citizen concerns and priorities about how the measures will affect their lives. These considerations—expert and lay—reflect different bodies of knowledge; broadly, technical or theoretical, and social or practical. It’s doubtful that successful policy can be derived from a consideration of only one of these categories of knowledge, nor is it clear that one is superior or should take priority over the other. There is, after all, little use in a technically perfect contact‐tracing system if citizens deem it morally anathema or logically incompatible with their lived experience.

Deliberative democracy or citizen science projects are venues at which these and other viewpoints can be mediated. They may not be appropriate policy‐making tools in the first and necessarily rushed stages of crisis management. But they are convenable at relatively short notice and can be conducted over a matter of days.

The biggest logistical task remains the selection process, which must deliver a representative sample of a given population, as well as a range of experts from different disciplines, with different perspectives on the matter in question. On an assembly model, the group (say, 100 people) will alternate between small group and plenary sessions; the former to ensure everyone who wants to contribute is able to, the latter to apprise the full group of the spectrum of opinion and priorities. Rather than *seeking* consensus—which risks the marginalisation of unpopular or minority voices—the aim should be to surface and circulate as many views as possible. This will enable citizens to speak to and be heard by the largest possible audience, and ensure policy makers are as familiar as they can be with the social and political topography that any resultant policy will need to be built upon.

Had the government convened a deliberative exercise shortly after imposing lockdown (either online or within social distancing parameters), the disproportionately high vulnerability of BAME communities—picked up eventually—might have been surfaced earlier. The lived testimony of these communities might also have shed light on the relative salience of their domestic situations, socio‐economic status, any common underlying health problems, or other structural disadvantages. Similar exercises could also, comfortably, be factored into policy‐making timetables directed at easing and eventually reversing Covid‐related lockdowns. I’ve mentioned immunity passporting. But efforts to shelter vulnerable communities for sustained periods, or stagger the reopening of schools, will need to reflect and respect the concerns of the communities affected (directly and contiguously) if they are to be effective and legitimate policies. Hosting deliberative events would moreover be a cheap and efficient alternative to live experimentation—that is, imposing policy on citizens and seeing what sticks (potentially reversing course or trying something different).

## Conclusion

Beyond the possibility of knowing more or knowing broader, deliberation and citizen science strengthen and promote the political value of public participation. Engaging the people due to be affected by policy is a sensible way to generate policy that reflects a *range* of interests—both lay and expert. But the upshot, whatever the policy specifics, is public participation itself; the fact that consent has been sought, and that citizens are acting and being treated like political agents—all of which relies on politicians engaging seriously with the process and outcomes of deliberative exercises.

Even during a public health crisis—perhaps then particularly—citizens, as well as their representatives and other political actors, have a role to play in democratic life. Deliberative and citizen science exercises can invigorate that life by improving the interaction between citizens, experts and politicians. In the present moment, these interventions could help develop and legitimise the government’s Covid strategy. But the same mechanisms could also serve democracy in whatever state of ‘normality’ we subsequently find ourselves. A permanent or semi‐permanent deliberative body with a rotating membership, mandated to convene on issues of national importance and report back to government via a select committee or something similar, would expand the scope for inclusivity and participation. Soon enough, citizens would either be current or ex‐participants or know someone (who resembled them) who had participated. And everybody could reasonably expect to be called upon at some point in the future. The so‐called deliberative turn—still more evident on the page than in the world—would then be a live and instituted aspect of our democratic culture.

## Notes

^1^
C. Offe, ‘Referendum vs. institutionalized deliberation: what democratic theorists can learn from the 2016 Brexit decisions’, *Dædalus, the Journal of the American Academy of Arts & Sciences*, vol. 146, no. 3, 2017, pp. 14–27.

^2^
See A. Gutmann and D. Thompson, *Why Deliberative Democracy?*, Princeton, Princeton University Press, 2004; J. Fishkin and J. Mansbridge, ‘Introduction’, *Dædalus, the Journal of the American Academy of Arts & Sciences*, vol. 146, no. 3, 2017, pp. 6–13; and J. Habermas, ‘Political communication in media society: does democracy still enjoy an epistemic dimension? The impact of normative theory on empirical research’, *Communication Theory*, vol. 16, no. 4, 2006, pp. 411–426.

^3^
J. Cohen, ‘Deliberation and democratic legitimacy’, in A. Hamlin and P. Pettit, eds., *The Good Policy: Normative Analysis of the State*, Oxford, Blackwell, 1989, pp. 17–34.

^4^
J. Fishkin, ‘Reviving deliberative democracy: reflections on recent experiments’, in S. Coleman, A. Przybylska and Y. Sintomer, eds., *Deliberation and Democracy: Innovative Processes and Institutions*, Frankfurt, Peter Lang, 2016, pp. 99–108.

^5^
D. M. Kahan, et al., ‘The polarizing impact of science literacy and numeracy on perceived climate change risks’, *Nature Climate Change*, vol. 2, 2012, pp. 732–735; and D. M. Kahan, ‘What is the “science of science communication”?’, *Journal of Science Communication*, vol. 14, no. 3, 2015, pp. 1–10.

^6^
S. Jasanoff, ‘Technologies of humility: citizen participation in governing science’, *Minerva*, vol. 41, 2003, pp. 223–244.

^7^
P. Weingart, ‘Scientific expertise and political accountability: paradoxes of science in politics’, *Science and Public Policy*, vol. 26, no. 3, 1999, pp. 151–161.

^8^
Jasanoff, ‘Technologies of humility’, pp. 229–231.

^9^
See K. Bäckstrand, ‘Scientisation vs. civic expertise in environmental governance: eco‐feminist, eco‐modern and post‐modern responses’, *Environmental Politics*, vol. 13, no. 4, 2004, pp. 695–714.

^10^
M. Hulme, *Why we Disagree about Climate Change: Understanding Controversy, Inaction and Opportunity*, Cambridge, Cambridge University Press, 2009, pp. 114–207.

^11^
B. Wynne, ‘Public participation in science and technology: performing and obscuring a political conceptual category mistake’, *East Asian Science, Technology and Society: an International Journal*, vol. 1, no. 1, 2007, pp. 99–110.

